# Two-year patient-related outcome measures (PROM) of primary ventral and incisional hernia repair using a novel three-dimensional composite polyester monofilament mesh: the SymCHro registry study

**DOI:** 10.1007/s10029-019-01924-w

**Published:** 2019-03-18

**Authors:** J. F. Gillion, M. Lepere, C. Barrat, O. Cas, A. Dabrowski, F. Jurczak, H. Khalil, C. Zaranis, The French Club Hernie Study Group, Marlène Antor, Mathieu Beck, Christophe Berney, Daniel Binot, Denis Blazquez, Alain Bonan, Jacques Bousquet, Hassan Demian, Vincent Dubuisson, Axèle Champault-Fezais, Philippe Chastan, Jean-Michel Chollet, Jean-Pierre Cossa, Sebastien Demaret, Thierry Delaunay, Farouk Drissi, Timothée Gérard Dugue, Gérard Fromont, Jean-François Gillion, Claude Jacquin, Marie-Véronique Launay-Savary, Patrick Ledaguenel, Marc Lepère, Denis Lépront, Nathalie Le Toux, Jean-Hughes Longeville, Eric Magne, Philippe Ngo, Olivier Oberlin, Xavier Pavis d’Escurac, Jean-Baptiste Putinier, Yohann Renard, Benoît Romain, Stéphane Roos, Marc Soler, Jean-Marc Thillois, Philippe Tiry, Romain Verhaeghe, Philippe Vu, Constantin Zaranis

**Affiliations:** 1 Hôpital Privé d’Antony, 1 rue Velpeau, 92160 Antony, France; 2 Clinique Saint Augustin, Nantes, France; 3 Hôpital J Verdier, Bondy, France; 4 Centre Médico-Chirurgical, Fondation Wallerstein, Arès, France; 5 Clinique de Saint-Omer, Saint-Omer, France; 6 Clinique mutualiste de l’estuaire, Saint-Nazaire, France; 7 Chu-Hôpitaux De Rouen, Rouen, France; 8 Clinique du Mail, La Rochelle, France

**Keywords:** Hernia repair, Ventral hernia, Incisional hernia, Abdominal hernia, Laparoscopy, Surgical mesh

## Abstract

**Purpose:**

This study examined patient-related outcome measures (PROMs) after repair of ventral primary or incisional hernias using Symbotex™ composite mesh (SCM), a novel three-dimensional collagen-coated monofilament polyester textile.

**Methods:**

Pre-operative, peri-operative, and post-operative data were obtained from the French “Club Hernie” registry with 12- and 24-month follow-up.

**Results:**

One-hundred consecutive patients (mean age 62.0 ± 13.7; 51% female) underwent repair of 105 hernias: primary (39/105, 37.1%, defect area 5.2 ± 5.6 cm^2^) and incisional (66/105, 62.9%, 31.9 ± 38.7.8 cm^2^). The mean BMI was 29.7 (± 5.6 kg/m^2^). American Society of Anesthesiologists classifications were I 39.4%, II 37.4% and III 23.2%. 75% had risk factors for healing and/or dissection. Of 38 primary repairs, 37 were completed laparoscopically (combined approach *n* = 1), and of 62 incisional hernia repairs, 40 were completed laparoscopically, and 20 by open repair (combined approach *n* = 2). Laparoscopic was quicker than open repair (36.2 ± 23.5 min vs. 67.4 ± 25.8, *p* < 0001). Before surgery, 86.3% of hernias were reported to cause discomfort/pain or dysesthesia. At 24 months (93 of 100 patients), 91 (97.8%) reported no lump and 81 (87.1%) no pain or discomfort. Of 91 patients, 86 (94.5%) rated their repair “good” or “excellent.” There were nine non-serious, surgeon-detected adverse events (ileus, *n* = 3; seroma, *n* = 6) and one hernia recurrence (6–12 months).

**Conclusions:**

Compared to baseline, open and laparoscopic surgery improved PROMs 24 months after primary and incisional hernia repair. Minimal complications and recurrence support the long-term efficacy of SCM.

## Introduction

Primary ventral and incisional hernias of the abdominal wall are common, pose significant medical issues, and are associated with an economic burden [1, 2]. They may be unsightly, cause pain, interfere with professional activities and have an impact on quality of life (QoL). While up to one-third of hernias is asymptomatic, surgical repair is generally recommended to reduce the risk of obstruction and strangulation [3].

The laparoscopic approach to primary ventral and incisional hernia repair is increasingly popular, offering the potential advantages of similar recurrence rates but fewer post-operative complications in selected patients [4]. A Cochrane Collaboration review of ten randomized controlled trials demonstrated that laparoscopic surgery was associated with fewer wound infections, improved cosmesis, and a shorter hospital stay [5]. Outcomes are also widely accepted as being improved by the incorporation of mesh in both open and laparoscopic procedures [6]. Incorporating mesh appears to offer advantages even for small defects such as primary umbilical hernias ≤ 4 cm in width [7].

Recurrence after surgery is influenced by several patient factors, including obesity, fitness, and defect size [3, 8]. Similarly, technical factors and the choice of mesh can affect outcomes after primary ventral or incisional repair. For example, recurrence rates are reported to be lower where there is mesh overlap of at least 5 cm and there is a high mesh area-to-defect area (*M*/*D*) ratio [9, 10].

Although the trend has moved away from mesh placement inside the abdominal cavity due to long-term complications related to adhesion formation, most laparoscopic ventral hernia repairs worldwide still employ an intraperitoneal onlay mesh (IPOM) technique. When using an intraperitoneal approach, reducing adhesion formation and prevention of damage to adjacent viscera are key aims [11]. Device manufacturers have sought to minimize this problem by including a continuous protective layer in their mesh designs. One early product was Parietex composite mesh (PCO, Covidien LP, Trevoux, France, a wholly owned subsidiary of Medtronic plc). First introduced in 1998, this multifilament polyester associated with a strong safety profile and minimal adhesion formation proved a popular repair option with over a million units sold to date [12]. Suggestions from surgeons regarding desired handling characteristics and memory shape resulted in a change from a multi- to a monofilament polyester-knitted structure and the development of Symbotex™ composite mesh (SCM; Covidien LP).

SCM is a novel three-dimensional monofilament polyethylene terephthalate (polyester) textile. On its visceral surface is a modified absorbable hydrophilic film consisting of a mixture of collagen and glycerol [13]. Other claimed design features include increased conformability, transparency, green centering marking, and a green flap, all designed to help visualize the fixation area and facilitate accurate position of the prosthesis against the abdominal wall.

Results, 12 months after laparoscopic or open mesh repair, suggest that the use of SCM is safe and effective [14]. The present study, an update of this registry study, aimed to examine outcomes 24 months after surgery with reference to factors which are important to patients, e.g., have their symptoms resolved compared to baseline and are they satisfied with the outcome of their treatment. The need to focus on patient-reported outcome measures (PROMs) is increasingly recognized [15]. From a surgical perspective, the researchers also sought to examine the effect of the *M*/*D* ratio on hernia recurrence [10]. Because primary ventral and incisional hernias have been shown to differ in terms of patient characteristics and post-operative complications, data are presented and analyzed by subgroup [16].

## Methodology

### Overview

This prospective study was performed by the Club Hernie, a group of approximately 40 French surgeons specialized in parietal surgery. An online de-identified, encrypted registry was established by the group in 2011 to prospectively record all consecutive inguinal and ventral hernia operations in real time, ahead of any outcomes being known. The registry is comprised of 164 input boxes with predefined questions and answers that were chosen by a medical advisory board before collection began. All answers must be selected to enable patient entry onto the registry which promoted a high completion rate. Standard, anonymized data capture includes pre-operative, peri-operative, and post-operative details.

All Club Hernie members are required to sign a quality charter promising to provide detailed information and full disclosure.

A proportion of participating surgeons employed Symbotex™ composite mesh (SCM) in primary ventral and incisional hernia repair. The present analysis focused on the first 100 adult subjects included in the registry who underwent laparoscopic or open ventral hernia repair using SCM between July 2014 and May 2015.

This investigation was registered on http://www.clinicaltrials.gov (NCT02206828). The French national ethics committee, the Comité de Protection des Personnes (CPP), was informed, and it subsequently issued a study waiver (CPP Sud Est III Lyon-File QH 15/2014) due to the registry nature of the study. Covidien LP provided technical support to the registry on a pro bono basis but did neither influence contents, patient results nor data interpretation.

### Study aims, objectives and endpoints

The aim of this observational registry study was to assess the short- and long-term clinical outcomes following the use of SCM in primary and incisional abdominal wall hernia surgeries by open or laparoscopic approach according to instructions for use.

The primary objectives (and endpoints) were to evaluate the incidence of peri-operative and post-operative complications, hernia recurrence and PROM during the first 2 years of follow-up. Secondary objectives (endpoints) included assessment of surgical techniques, mesh fixation and handling, operative time, surgeon satisfaction and length of stay. Issues of importance to patients included QoL, individual satisfaction and pain assessment.

### Patients and sample size

Consecutive patients ≥ 18 years of age undergoing elective or emergency primary ventral and/or incisional hernia repair were included irrespective of the size and complexity of their defects. There were no exclusion criteria. The chosen sample size of 100 was based on previous experience using PCO mesh, which suggested that a recurrence rate below 6% could be expected after open or laparoscopic ventral hernia repair, with a standard 95% confidence interval and an estimated 15% loss to follow-up [17, 18]. Fitness for operation was assessed based on American Society of Anesthesiologists (ASA) physical status scores [19].

During a pre-operative visit, the responsible surgeon gave all patients a written notice informing them about the nature and purpose of the study. Because of the CPP waiver, completion of an informed consent form was not required.

### Operative treatment

Whether patients underwent laparoscopic or open repair of their primary ventral or incisional hernia using SCM was at the discretion of the surgeon. Similarly, the type of fixation was not imposed but recorded. The mesh was trimmed if necessary while ensuring sufficient overlap to cover the adjacent edges of the defect. An overlap greater than 5 cm has previously been shown to reduce the rate of recurrence [9]. Skin-to-skin operating time and time required to fix the mesh were recorded, along with surgeons’ views on various aspects of the procedure.

### Hernia defect and mesh size

Dimensions of the hernia defects (cm^2^) were calculated using a circular area formula for primary hernias and an elliptical formula for incisional hernias [20]. A similar formula was used for mesh size. Based on these dimensions, *M*/*D* ratios were calculated.

### Choice of Symbotex composite mesh

Surgeons were permitted to choose an appropriate size and shape from the three available types of SCM: a flat sheet without sutures (SYM), a flat sheet with prepositioned sutures (SYM-F), and a flat sheet with an open textile flap (SYM-OS) designed to aid placement and fixation.

### Follow-up

**Fig. 1:**
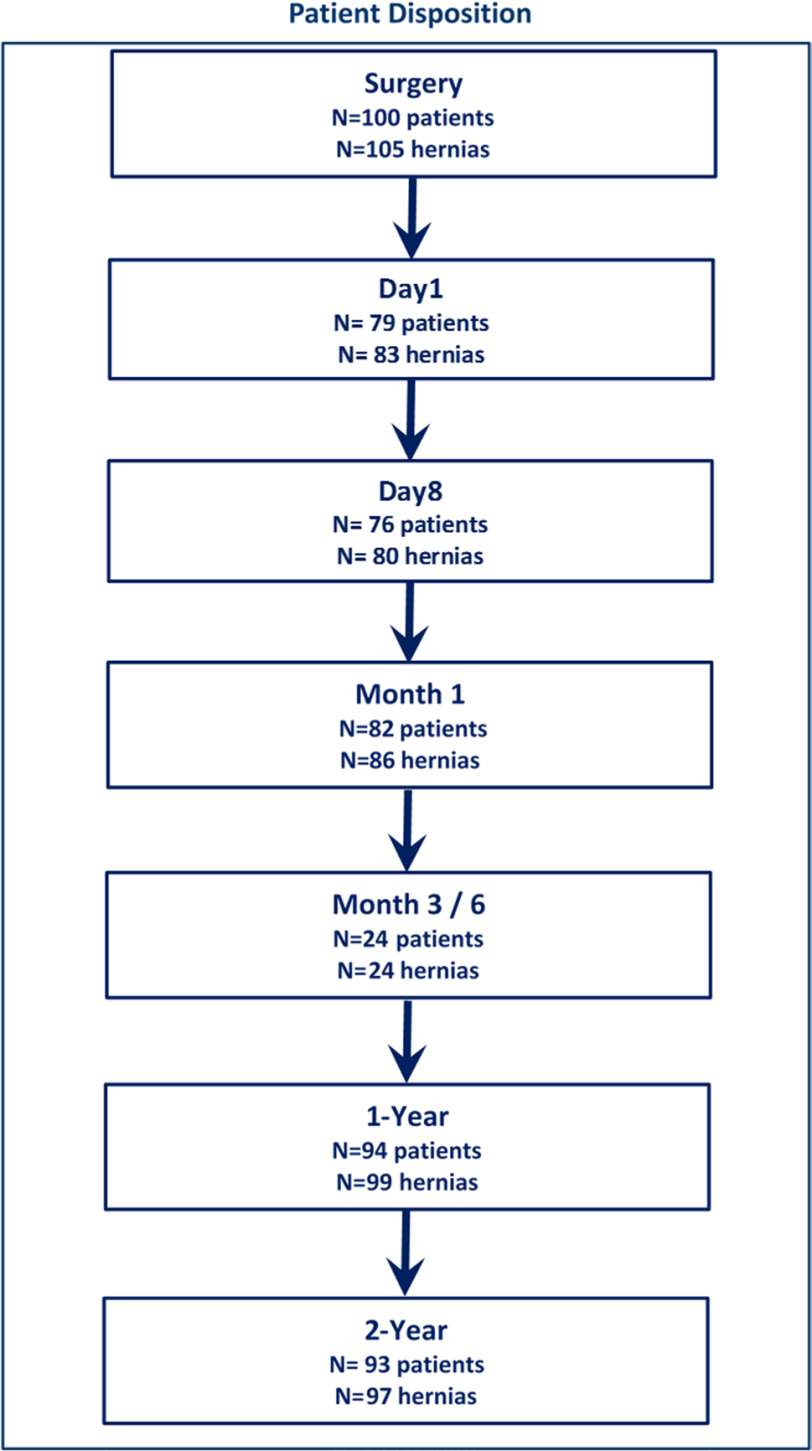
Study flow chart. Schedule of patient follow-up and number involved at each time point

Patients were followed up on at set points after surgery (Fig. 1). Pain assessment was performed by a nurse on the day of surgery, on Day 1, and on Day 8, either directly or by telephone if the patient had been discharged. Additional pain assessment was performed by the surgeon during the patient’s 1-month review appointment. All patients experiencing symptoms (e.g., pain or a bulge) were advised to attend a further follow-up appointment at 3 months. Follow-up at the end of year 1 and year 2 involved a detailed QoL questionnaire administered via telephone by a Club Hernie clinical research assistant acting independently of the operating surgeon [21]. The information was entered verbatim in the registry, without interpretation or adjustment.

Those patients from whom a response could not be elicited after five attempts were considered lost to follow-up to minimize non-response bias.

### Data presentation and analysis

Descriptive data per patient, hernia, and surgeon are presented numerically, in terms of means, standard deviations, and ranges. Missing values are mentioned in the relevant tables. Categorical variables are presented as frequencies and percentages of the number of recorded entries. A minority of patients had more than one hernia; where both types of hernia existed, their classification as primary or incisional was at the surgeon’s discretion.

Statistical analysis was performed using Minitab v15.0 and SAS 9.2. Comparative group analysis was performed using Student’s *t* test and Wilcoxon’s rank-sum test for non-parametric data. Qualitative data were examined using Pearson’s chi-squared test and Fisher’s exact test, as appropriate.

Pain was assessed at intervals during the study using a visual analog scale (VAS). Comparison of paired data was performed using Student’s *t* test and Wilcoxon’s signed-rank test.

## Results

### Patient demographics

One-hundred consecutive patients (mean age 62.0 ± 13.7; 51% female) who entered into the Club Hernie registry between July 2014 and May 2015 underwent repair of 105 hernias. 39/105 were primary (37.1%, defect area 5.2 ± 5.6 cm^2^) and the remaining 66/105 were incisional (62.9%, 31.9 ± 38.7 cm^2^). The mean BMI was 29.7 (± 5.6 kg/m^2^). ASA classifications were I 39.4%, II 37.4% and III 23.2%. 75% of patients had risk factors for healing and/or dissection. Patients with an incisional hernia were older (mean age 63.4 ± 13.1 years vs. 58.1 ± 13.8 years; *p* = 0.026) and tended to report a hernia history (32/62, 51.6% vs. 4/38, 10.5%; *p* < 0.0001; Table 1).

**Table 1:** Patient demographics, including body mass index (BMI), smoking status, American Society of Anesthesiologists (ASA) physical status score, hernia history, risk factors related to surgical dissection and healing (*n* = 100)

Criteria	Overall patients (*n* = 100)	Primary hernia patients (*n* = 38)	Incisional hernia patients (*n* = 62)	*p* value
Gender				
Male	49 (49.0)	22 (57.9)	27 (43.5)	0.1640
Female	51 (51.0)	16 (42.1)	35 (56.5)	
Age (years)	62 (13.7)	58.1 (13.8)	63.4 (13.1)	0.0260
BMI (kg/m^2^)	29.7 (5.6)	30.4 (6.1)	29.3 (5.2)	0.377
Missing data	1	1	0	
Smoking Status				
Non-smoker (never smoked or stopped ≥ 12 months)	83 (84.7)	33 (86.8)	50 (83.3)	0.631
Smoker (current, recent, regular or occasional smoker)	15 (15.3)	5 (13.2)	10 (16.7)	
Missing data	2	0	2	
ASA classification				
Class I	39 (39.4)	24 (64.9)	15 (24.2)	0.0003
Class II	37 (37.4)	8 (21.6)	29 (46.8)	
Class III	23 (23.2)	5 (13.5)	18 (29.0)	
Class IV/V	0 (0.0)	0 (0.0)	0 (0.0)	
Missing data	1	1	0	
Hernia history				
None	64 (64.0)	34 (89.5)	30 (48.4)	< 0.0001
Previous history (one or more)	36 (36.0)	4 (10.5)	32 (51.6)	
Risk factors related to surgical dissection				
No	31 (31.3)	21 (56.8)	10 (16.1)	< 0.0001
Yes (one or more)	68 (68.7)	16 (43.2)	52 (83.9)	
Missing data	1	1	0	
Risk factors related to healing				
No	65 (66.3)	30 (78.9)	35 (58.3)	0.0354
Yes (one or more)	33 (33.7)	8 (21.1)	25 (41.7)	
Anticoagulant treatment or spontaneous coagulation/bleeding disorder	18 (18.4)	4 (10.5)	14 (23.7)	
Chemotherapy/immunosuppressive treatment	8 (8.2)	1 (2.6)	7 (11.9)	
Diabetes	7 (7.1)	0 (0.0)	7 (11.9)	
Corticosteroids	4 (4.1)	0 (0.0)	4 (6.8)	
Other (unspecified)	9 (9.2)	3 (7.9)	6 (10.0)	
Missing data	2	0	2	
Risk factors related to healing and/or surgical dissection				
No	25 (25.0)	18 (47.4)	7 (11.3)	< 0.0001
Yes (one or more)	75 (75.0)	20 (52.6)	55 (88.7)	

Results are expressed in n (%) for categorical data and mean (SD) for quantitative data; *p* value < 0.05 is significant

*BMI* Body Mass Index, *ASA* American Society of Anesthesiologists physical status score

In patients classified as having an incisional hernia, these defects were predominantly situated in the midline (*n* = 47, epigastric *n* = 15, peri-umbilical *n* = 14, sub-umbilical *n* = 6, supra-pubic *n* = 1, mixed *n* = 12) or laterally (*n* = 12, sub-costal *n* = 3, flank *n* = 3, iliac *n* = 4, lumbar *n* = 2). Three patients were reported as having both midline and lateral hernias. By comparison, in patients with a primary hernia, 30/38 were classified as having an umbilical/sub-umbilical defect. Other types were epigastric (*n* = 6), Spigelian (*n* = 1), and mixed (umbilical/sub-umbilical and epigastric *n* = 1).

### Pre-operative symptoms

Information on pre-operative symptoms was available for 102 of 105 hernias (missing values *n* = 3; Table 2). While 7 of 102 hernias (6.9%; primary *n* = 2; incisional *n* = 5) were asymptomatic, most operated hernias caused discomfort, pain, or dysesthesia (88/102, 86.3%). Seven hernias (6.9%) were described as strangulated. In five, the contents of the hernias were reducible by taxis, and in one, the hernia was strangulated without bowel occlusion. In the remaining patient, there was evidence of strangulation and occlusion.

**Table 2:** Hernia details, including symptoms and dimensions (*n* = 105)

Criteria	Overall hernias (*n* = 105)	Primary hernias (*n* = 39)	Incisional Hernias (*n* = 66)	*p* value
Multisite hernia				
No	83 (79.8)	34 (89.5)	49 (74.2)	0.0624
Yes	21 (20.2)	4 (10.5)	17 (25.8)	
Missing data	1	1	0	
Hernia symptoms				
Asymptomatic hernia	7 (6.9)	2 (5.1)	5 (7.9)	
Discomfort/pain or pre-operative dysesthesia	88 (86.3)	34 (87.2)	54 (85.7)	
Hernia reducible by taxis	5 (4.9)	2 (5.1)	3 (4.8)	
Strangulated hernia without occlusion	1 (1.0)	1 (2.6)	0 (0.0)	
Strangulated hernia with occlusion	1 (1.0)	0 (0.0)	1 (1.6)	
Missing data	3	0	3	
Incarcerated hernia				
No	71 (68.9)	30 (76.9)	41 (64.1)	0.1713
Yes	32 (31.1)	9 (23.1)	23 (35.1)	
Missing data	2	0	2	
Hernia defect width (cm)	3.9 (2.5)	2.3 (1.0)	4.9 (2.6)	< 0.0001
Missing data	1	0	1	
Hernia defect length (cm)	5.0 (4.1)	2.5 (1.2)	6.5 (4.5)	< 0.0001
Missing data	1	0	1	
Hernia defect area (cm^2^)	21.9 (33.3)	5.2 (5.6)	31.9 (38.7)	< 0.0001
Missing data	1	0	1	
Mesh positioning time (minutes)	9.4 (6.3)	8.5 (5.6)	9.9 (6.6)	0.2652
Missing data	3	1	2	
Mesh area to defect area ratio (*M*/*D*)	35.1 (46.4)	55.7 (64.1)	22.7 (24.8)	< 0.0001
Missing data	1	0	1	

Results are expressed in n (%) for categorical data and mean (SD) for quantitative data; *p* value < 0.05 is significant

### Hernia details

A total of 105 hernias were treated. 39 of 105 (37.1%,) hernias were classified as primary, and 66 of 105 (62.9%) as incisional. Four patients had more than one separate hernia: two patients had two incisional hernias, one patient had both a primary and incisional hernia (classified as incisional), and one patient had three incisional hernias.

At operation, defects appeared to be multifocal in 21 of 104 hernias (20.2%; unifocal *n* = 83, 79.8%; missing value *n* = 1; Table 2). Thirty-two of 103 hernias were reported as incarcerated (31.1%; not incarcerated *n* = 71, 68.9%; missing values *n* = 2).

Primary hernia defects (*n* = 39) were found to be smaller than incisional hernia defects (*n* = 65, missing value *n* = 1) in width (2.3 ± 1.0 cm vs. 4.9 ± 2.6 cm; *p* < 0.0001), length (2.5 ± 1.2 cm vs. 6.5 ± 4.5 cm; *p* < 0.0001), and total defect area (5.2 ± 5.6 cm^2^ vs. 31.9 ± 38.7 cm^2^; *p* < 0.0001; Table 2).

### Surgical approach

Surgical repair using SCM was performed at 14 French centers. All procedures were under general anesthetic. Three operations were classified as an emergency (primary *n* = 1; incisional *n* = 2). All but three operations were considered “clean” (“clean contaminated”: primary *n* = 1; incisional *n* = 2). In 37 of 38 patients with a primary hernia, repair was completed laparoscopically, with one case requiring a combined open and laparoscopic approach (Table 3). In 40 of 62 patients (64.5%) with an incisional hernia, the operation was planned and completed laparoscopically. Similarly, in 20 of 62 (32.3%) patients, the operation was planned and completed as an open procedure. 2 of 62 incisional hernia repairs involved a hybrid open and laparoscopic approach.

**Table 3:** Summary of operative data by hernia type, including whether an emergency or elective procedure, Altemeier classification, type of anesthesia administered, surgical approach, use of antibiotic prophylaxis, total operating time and time for mesh placement, the mesh/defect ration (*M*/*D*) and hospital stay (*n* = 100)

Criteria	Overall patients (*n* = 100)	Primary hernias patients (*n* = 38)	Incisional hernias patients (*n* = 62)	*p* value
Emergency surgery				
No	97 (97.0)	37 (97.4)	60 (96.8)	1.0000
Yes	3 (3.0)	1 (2.6)	2 (3.2)	
Altemeier classification				
Clean	97 (97.0)	37 (97.4) (89.5)	60 (96.8)	1.0000
Clean contaminated	3 (3.0)	1 (2.6)	2 (3.2)	
Surgical approach				
Open (laparotomy)	20 (20.0)	0 (0.0)	20 (32.3)	< 0.0001
Laparoscopic	77 (77.0)	37 (97.4)	40 (64.5)	
Hybrid open/laparoscopic	3 (3.0)	1 (2.6)	2 (3.2)	
Antibiotic prophylaxis				
No	15 (15.0)	11 (28.9)	4 (6.5)	0.0022
Yes	85 (85.0)	27 (71.1)	58 (93.5)	
Single dose	84 (84.0)	27 (71.1)	57 (91.9)	
Prolonged	1 (1.0)	0 (0.0)	1 (1.6)	
Operative time (min)	43.4 (27.3)	30.9 (21.8)	50.9 (27.7)	0.0004
Missing data	4	2	2	
Hospital stay (days)	2.5 (2.3)	0.9 (1.1)	2.9 (2.1)	< 0.0001
Missing data	8	2	6	

Results are expressed in *n* (%) for categorical data and mean (SD) for quantitative data; *p* value < 0.05 is significant

Larger incisional hernias tended to be operated on via an open approach (laparoscopic repair mean defect size 17.3 cm^2^ ± 20.2 vs. open repair 59.5 cm^2^ ± 50.5; *p* < 0.0001), took longer to perform, and involved patients spending more time in the hospital (see below).

### Operative duration, time for mesh placement, and hospital stay

Overall, laparoscopic repair was quicker than open repair (36.2 ± 23.5 min vs. 67.4 ± 25.8, *p* < 0.0001; Table 4) and laparoscopy patients went home earlier (1.6 ± 1.6 hospital days vs. 4.7 ± 2.0, *p* < 0.0001). Similarly, repairing an incisional hernia laparoscopically took less time than an open repair (41.6 ± 24.0 min vs. 67.4 ± 25.8; *p* < 0.0001; Table 4) and was associated with a shorter inpatient stay (2.2 ± 1.7 hospital days vs. 4.7 ± 2.0, *p* < 0.0001). A total of 20 procedures were ambulatory (primary 15/36 vs. incisional 5/56; *p* < 0.0001; missing *n* = 8).

**Table 4:** Summary of operative data by surgical approach (laparoscopic, open, combined open/laparoscopic): total operating time, time for mesh placement and hospital stay (*n* = 100)

Criteria	Laparoscopic approach patients (*n* = 77)	Open approach patients (*n* = 20)	Hybrid open/lap approach patients (*n* = 3)	*p* value
Hernia type				
Primary hernia	37 (48.1)	0 (0.0)	1 (33.3)	< 0.0001
Incisional hernia	40 (51.9)	20 (100.0)	2 (66.7)	
Operative time (min)	36.2 (23.5)	67.4 (25.8)	68.3 (32.1)	< 0.0001
Missing data	3	1	0	
Operative time for primary hernia patients	30.3 (21.7)	n/a	55 (0.0)	n/a
Missing data	2	0	0	
Operative time for Incisional hernia patients	41.6 (24.0)	67.4 (25.8)	75.0 (42.4)	< 0.0001
Missing data	0	1	0	
Hospital stay (days)	1.6 (1.6)	4.7 (2.0)	2.9 (2.1)	< 0.0001
Missing data	5	3	0	

Results are expressed in n (%) for categorical data and mean (SD) for quantitative data; *p* value < 0.05 is significant

*p* values are for laparoscopic vs. open approach

All meshes were positioned intraperitoneally including one partially inserted in the preperitoneal space. The mean time required to position the mesh was 9.4 ± 6.3 min (laparoscopic 8.6 ± 5.5 vs. open 10.1 ± 5.8; Table 2). 83 (39 primary and 44 incisional) of the 105 hernias were repaired laparoscopically. Fascial closure was achieved in 26 of these 83 cases (31%), in 9 of 39 primary (23%), and 17 of 44 incisional (39%) hernias (*χ*^2^ = 2,33). Fascial closure was also more frequent in larger defects (15.3 cm^2^ ± 10.3 vs. 4.7 cm^2^ ± 2.8; *p* = 0.0011). The mesh fixation consisted of stapling using absorbable (61 cases) and non-absorbable (22 cases) tackers or with sutures in 11 cases. Double crown stapling was used in 17 cases. Glue was not used. Mesh overlap ≥ 5 cm was achieved in 76 of 80 laparoscopic repairs (95.0%; excludes hybrid repairs) and 16 of 22 open repairs (72.7%).

### Mesh area-to-defect area ratios

Information on *M*/*D* ratios was collected in 104 of 105 hernias (Table 3). The mean *M*/*D* ratio was 55.7 (± 64.1) and 22.7 (± 24.8) for primary and incisional hernia defects, respectively (*p* < 0.0001). The ratio was larger for laparoscopic repairs than open repairs (39.9 ± 49.0 vs. 12.0 ± 15.4, *p* < 0.0001; combined open/laparoscopic 77.1 ± 75.1).

### Symbotex composite mesh handling characteristics

Flexibility of the mesh and ease of insertion were rated as “satisfying” in 102 hernia repairs (Tables 5, 6). Similarly, in 80 hernias, the mesh was reported as “satisfying” to trim (not applicable or unknown *n* = 20). Shape memory was also generally considered “satisfying,” as was the low visceral attachment. In two hernia repairs, shape memory was rated as “unsatisfying” (2.1%). Several features of the mesh were identified as beneficial to successfully completing operations (Tables 5, 7).

**Table 5:** Summary of the defect size and *M*/*D* ratio according to fascial closure and surgical approach

Overall hernias (*n* = 105 hernias)	Laparoscopic approach		Open approach	
	Closed fascia (*n* = 26 hernias)	Unclosed fascia (*n* = 57 hernias)	Closed fascia (*n* = 9 hernias)	Unclosed fascia (*n* = 13 hernias)
Defect area (cm^2^)	16.4 (12.1)	9.7 (17.5)	61.5 (61.5)	58.1 (43.9)
Missing data	0	1	0	0
*p* value (closed vs. unclosed)	0.0009		0.6159	
Ratio mesh size/defect area	25.4 (22.9)	48.7 (57.1)	19.7 (22.3)	6.7 (3.1)
Missing data	0	1	0	0
*p* value (closed vs. unclosed)	0.0018		0.2699	

Primary hernias (*n* = 39 hernias)	Closed fascia (*n* = 10 hernias)	Unclosed fascia (*n* = 29 hernias)	Closed fascia (*n* = 0)	Unclosed fascia (*n* = 0)
Defect area (cm^2^)	8.5 (9.4)	4.1 (3.0)	n/a (primary hernias were all repaired laparoscopically)	
Missing data	0	0		
*p* value (closed vs. unclosed)	0.1456			
Ratio mesh size/defect area	39.5 (28.7)	61.4 (72.0)		
Missing data	0	0		
*p* value (closed vs. unclosed)	0.4076			

Results for incisional hernias (*n* = 66 hernias)	Closed fascia (*n* = 16 hernias)	Unclosed fascia (*n* = 28 hernias)	Closed fascia (*n* = 9 hernias)	Unclosed fascia (*n* = 13 hernias)
Defect area (cm^2^)	21.3 (11.2)	15.7 (23.8)	61.5 (61.5)	58.1 (43.9)
Missing data	0	1	0	0
*p* value (closed vs. unclosed)	0.0071		0.6159	
Ratio mesh size/defect area	16.6 (12.8)	35.0 (30.8)	19.7 (22.3)	6.7 (3.1)
Missing data	0	1	0	0
*p* value (closed vs. unclosed)	0.0055		0.2699	

**Table 6:** Surgeons’ views of Symbotex mesh handling characteristics: mesh flexibility, ease of mesh trimming, ease of mesh insertion, mesh shape memory and low visceral attachment

Criteria	Overall hernias (*n* = 105)	Primary hernias (*n* = 39)	Incisional hernias (*n* = 66)	*p* value
Mesh flexibility				
Satisfying	102 (100.0)	38 (100.0)	64 (100.0)	1.0000
Unsatisfying	0 (0.0)	0 (0.0)	0 (0.0)	
n/a	0	0	0	
Missing data	3	1	2	
Ease of mesh trimming				
Satisfying	80 (100.0)	31 (100.0)	49 (100.0)	1.0000
Unsatisfying	0 (0.0)	0 (0.0)	0 (0.0)	
n/a	20	6	14	
Missing data	5	2	3	
Ease of mesh insertion				
Satisfying	102 (100.0)	38 (100.0)	64 (100.0)	1.0000
Unsatisfying	0 (0.0)	0 (0.0)	0 (0.0)	
n/a	0	0	0	
Missing data	5	1	3	
Mesh shape memory				
Satisfying	94 (97.9)	36 (100.0)	58 (96.7)	0.5260
Unsatisfying	2 (2.1)	0	2 (3.3)	
n/a	2	0	2	
Missing data	7	3	4	
Low visceral attachment				
Satisfying	92 (100.0)	35 (100.0)	57 (100.0)	1.0000
Unsatisfying	0 (0.0)	0 (0.0)	0 (0.0)	
n/a	9	2	7	
Missing data	4	2	2	

Results are expressed in *n* (%); *p* value < 0.05 is significant

*n*/*a* is for “not-applicable” (surgeon did not have opportunity to evaluate the mesh property)

**Table 7:** Features of Symbotex mesh found by surgeons to assist primary and incisional hernia repair

Criteria	Overall hernias (*n* = 105)	Primary hernias (*n* = 39)	Incisional hernias (*n* = 66)	*p* value
Mesh transparency aids placement	74 (74.0)	26 (72.2)	48 (75.0)	0.7610
Missing data	5	3	2	
Mesh marking assists placement	68 (68.0)	28 (77.8)	40 (62.5)	0.1160
Missing data	5	3	2	
Prosthesis shape memory assists placement	53 (53.0)	14 (38.9)	39 (60.9)	0.0340
Missing data	5	3	2	
Mesh easy to reposition	51 (51.0)	16 (44.4)	35 (54.7)	0.3250
Missing data	5	3	2	
Mesh does not switch during fixation/stapling	43 (43.0)	14 (38.9)	29 (45.3)	0.5330
Missing data	5	3	2	
Mesh conformability to the anatomy during placement is useful	34 (34.0)	16 (44.4)	18 (28.1)	0.0980
Missing data	5	3	2	
No requirement to orient mesh is useful	32 (32.0)	16 (44.4)	16 (25.0)	0.0460
Missing data	5	3	2	

Results are expressed in *n* (%); *p* value < 0.05 is significant

### Follow-up and adverse events

Patients underwent follow-up for a mean of 719.2 days (23.9 months; Fig. 1). There were no deaths nor serious adverse events reported during this study (available patient data at end of year 1 *n* = 94, year 2 *n* = 93).

In total, ten adverse events (primary hernia 4/38 patients, 10.5%; incisional hernia 6/62, 9.7%) were reported in the course of 24-month follow-up. During another surgical procedure, one sub-costal incisional hernia was noted 6–12 months after midline incisional hernia repair using SCM. Though likely to be a new hernia following previous hepatic surgery, this defect was classified as a “recurrence”. According to the operating surgeon, the small, asymptomatic defect does not at present require a reintervention.

Three patients experienced a mild, transitory ileus after incisional hernia repair (peri-operative 2/100; 2–4 weeks post-operative 1/82). It is unknown whether these experiences were related to intra-operative adhesiolysis, mesh placement or the implant itself. Similarly, six patients were noted to have had a seroma (peri-operative 1/100; 2–4 weeks post-operative 5/82, 6.1%). In four, the seroma followed a primary hernia repair, and in two, an incisional hernia repair. These seromas were not considered mesh related. One seroma required aspiration, whereas all others resolved spontaneously.

### Patient satisfaction

At 12 months after surgery, 94 patients took part in a self-administered questionnaire. At 24 months, 93 patients were responding to the questionnaire. Earlier research demonstrated that post-operative pain scores improved progressively with time [14]. At 12 months, patients’ average VAS score was 3; by the second year, this had reduced to 2.

Patient satisfaction is shown in Table 8. The proportion of patients rating their outcome as “good” or “excellent” rose slightly with time (year 1 88.3% vs. year 2 94.5%) while the proportion considering their outcome “medium” or “bad” fell from 11.7% at year 1 to 5.5% at year 2. There was no significant difference between ratings by patients undergoing primary and incisional hernia repair.

**Table 8:** Patient-related outcome measures (PROM) at the end of year 1 and year 2: overall satisfaction, perceived abdominal wall firmness, pain/discomfort, location of any symptoms, timing of symptoms, frequency of symptoms, effect of symptoms on activity and comparison of symptom nuisance before and after surgery

Criteria	Response at 1 year				Response at 2 year			
	Overall patients (*n* = 100)	Primary hernias patients (*n* = 38)	Incisional hernias patients (*n* = 62)	*p* value	Overall patients (*n* = 100)	Primary hernias patients (*n* = 38)	Incisional hernias patients (*n* = 62)	*p* value
Q1: Does your abdominal wall seem firm?								
No	4 (4.3)	0 (0.0)	4 (6.9)	0.2942	2 (2.2)	0 (0.0)	2 (3.4)	0.5255
Yes	90 (95.7)	36 (100.0)	54 (93.1)		91 (97.8)	35 (100.0)	56 (96.6)	
Missing data	6	2	4		7	3	4	
Q2: Do you feel a lump?								
No	87 (93.5)	36 (100.0)	51 (89.5)	0.0780	91 (97.8)	35 (100.0)	56 (96.6)	0.5250
Yes	6 (6.5)	0 (0.0)	6 (10.5)		2 (2.2)	0 (0.0)	2 (3.4)	
Yes, on operated area	2 (2.2)	0 (0.0)	2 (3.5)		0 (0.0)	0 (0.0)	0 (0.0)	
Yes, on midline area	3 (3.2)	0 (0.0)	3 (5.2)		2 (2.2)	0 (0.0)	2 (3.4)	
Yes, elsewhere	1 (1.1)	0 (0.0)	1 (1.7)		0 (0.0)	0 (0.0)	0 (0.0)	
Missing data	7	2	5		7	3	4	
Q3: Do you feel any pain or discomfort?								
No	71 (75.5)	29 (80.6)	42 (72.4)	0.3560	81 (87.1)	32 (91.4)	49 (84.5)	0.3470
Yes	23 (24.5)	7 (19.4)	16 (27.6)		12 (12.9)	3 (8.6)	9 (15.5)	
Yes, mild pain or discomfort	13 (13.8)	6 (16.7)	7 (12.1)		5 (5.4)	1 (2.9)	4 (6.9)	
Yes, moderate pain (no analgesia required)	8 (8.5)	1 (2.8)	7 (12.1)		6 (6.5)	2 (5.7)	4 (6.9)	
Yes, severe pain (analgesia required)	1 (1.1)	0 (0.0)	1 (1.7)		0 (0.0)	0 (0.0)	0 (0.0)	
Yes, loss of sensitivity	0 (0.0)	0 (0.0)	0 (0.0)		1 (1.1)	0 (0.0)	1 (1.7)	
Yes, other (description is missing)	1 (1.1)	0 (0.0)	1 (1.7)		0 (0.0)	0 (0.0)	0 (0.0)	
Missing data	6	2	4		7	3	4	
Q4 through Q8 are restricted to patients feeling pain or discomfort								
Q4: Where are the symptoms located?								
Operated hernia	5 (21.7)	2 (28.6)	3 (18.8)		0 (0.0)	0 (0.0)	0 (0.0)	
Midline	17 (73.9)	5 (71.4)	12 (75.0)		11 (91.7)	2 (66.7)	9 (100.0)	
Controlateral	1 (4.3)	0 (0.0)	1 (6.3)		0 (0.0)	0 (0.0)	0 (0.0)	
Testicle region	0 (0.0)	0 (0.0)	0 (0.0)		1 (8.3)	1 (33.3)	0 (0.0)	
Missing data	0	0	0		0	0	0	
Q5: When do you feel these symptoms?								
During lifting, coughing	0 (0.0)	0 (0.0)	0 (0.0)		0 (0.0)	0 (0.0)	0 (0.0)	
During other types of efforts	11 (50.0)	2 (33.3)	9 (56.3)		3 (30.0)	1 (50.0)	2 (25.0)	
At the end of the day	1 (4.5)	1 (16.7)	0 (0.0)		0 (0.0)	0 (0.0)	0 (0.0)	
At any time	10 (45.5)	3 (50.0)	7 (43.8)		7 (70.0)	1 (50.0)	6 (75.0)	
Missing data	1	1	0		2	1	1	
Q6: How often do you feel these symptoms?								
Rarely	8 (38.1)	2 (33.3)	6 (40.0)		5 (50.0)	1 (50.0)	4 (50.0)	
Several times a week	9 (42.9)	3 (50.0)	6 (40.0)		1 (10.0)	0 (0.0)	1 (12.5)	
Several times a day	1 (4.8)	0 (0.0)	1 (6.7)		2 (20.0)	1 (50.0)	1 (12.5)	
Often	1 (4.8)	0 (0.0)	1 (6.7)		2 (20.0)	0 (0.0)	2 (25.0)	
Thoughout the day/all day long	2 (9.5)	1 (16.7)	1 (6.7)		0 (0.0)	0 (0.0)	0 (0.0)	
Missing data	2	1	1		2	1	1	
Q7: Effect of symptoms on activity								
Do not hinder your activities	14 (66.7)	4 (66.7)	10 (66.7)		7 (70.0)	1 (50.0)	6 (75.0)	
Allow you to pursue activities at a slower pace	7 (33.3)	2 (33.3)	5 (33.3)		3 (30.0)	1 (50.0)	2 (25.0)	
Cause temporary interruption of your activities	0 (0.0)	0 (0.0)	0 (0.0)		0 (0.0)	0 (0.0)	0 (0.0)	
Prevent certain activities	0 (0.0)	0 (0.0)	0 (0.0)		0 (0.0)	0 (0.0)	0 (0.0)	
Missing data	2	1	1		2	1	1	
Q8: Nuisance of pain/discomfort is								
< Hernia discomfort before surgery	18 (81.8)	5 (71.4)	13 (86.7)		7 (63.6)	2 (66.7)	5 (62.5)	
> Hernia discomfort before surgery	4 (18.2)	2 (28.6)	2 (13.3)		4 (36.4)	1 (33.3)	3 (37.5)	
Missing data	1	0	1		1	0	1	
Q9: Patient satisfaction								
Excellent	10 (10.6)	6 (16.7)	4 (6.9)	0.5262	7 (7.7)	1 (2.9)	6 (10.5)	0.2693
Good	73 (77.7)	27 (75.0)	46 (79.3)		79 (86.8)	32 (94.1)	47 (82.5)	
Medium	7 (7.4)	2 (5.6)	5 (8.6)		3 (3.3)	0 (0.0)	3 (5.3)	
Bad	4 (4.3)	1 (2.8)	3 (5.2)		2 (2.2)	1 (2.9)	1 (1.8)	
Missing data	6	2	4		9	4	5	

Results are expressed in n (%); *p* value < 0.05 is significant

At year 1, 90 of 94 (95.7%) considered their abdominal wall “firm,” compared with 91 of 93 (97.8%) at year 2 (Table 8). Of 93 patients, 6 (all in the incisional hernia group) reported a lump at year 1, compared with 2 (both in the incisional hernia group) at year 2.

In response to the question “Do you feel pain or discomfort?”, 71 of 94 patients said “No” (75.5%) at year 1, compared with 81 of 93 (87.1%) at year 2. Of the 12 patients (12.9%) with symptoms at year 2, 5 (5.4%) described these symptoms as “discomfort,” 6 (6.5%) as “moderate pain not requiring analgesia”, and 1 (1.1%) as “loss of sensitivity.” Further details are provided in Table 8.

## Discussion

The results of this observational study of 100 patients extracted from the Club Hernie registry show that patients with a primary or incisional ventral hernia generally experienced discomfort/pain or dysesthesia before surgery. Follow-up at 24 months after a laparoscopic or open repair found that nearly 90% of patients were pain and discomfort free, and almost 100% were unable to feel a lump; most grading their result as ‘good’ or ‘excellent’.

Using SCM to repair primary and incisional abdominal hernias was found to be safe and effective. Early post-operative complications were mild (Clavien–Dindo ≤ 2) and non-life threatening: [22]. There was one suspected hernia recurrence detected during another procedure 6–12 months after surgery which is not at present considered to require treatment. On inspection, this was found to be a sub-costal defect which was not originally treated in the study. Notably, the study population was unselected, generally middle aged or elderly, and overweight, and had risk factors potentially complicating surgical dissection and post-operative recovery. Surgeons reported that the mesh generally handled to their satisfaction during both laparoscopic and open approaches.

Hauters et al. stated that “The ultimate measure of hernia repair remains its recurrence rate” [10]. Nearly two-thirds of hernias in the present study were classified as incisional. Whereas meta-analyses of laparoscopic and open incisional ventral hernia repairs in 1,003 patients have shown recurrence rates of 6.99 and 4.82%, respectively [23], Chelala et al. have suggested that the true recurrence following laparoscopic repair of wider defects is around 12%, with values after open repair in exceptional circumstances reaching 32% [13]. This study’s early finding of only one possible hernia recurrence in a complex patient who had undergone previous liver surgery is encouraging but needs to be put into context. Patients were not examined at 24 months, nor did they undergo objective tests such as abdominal ultrasound scanning. As hernias can recur at any point during follow-up, later defects may be discovered in other patients in the present cohort [4, 5, 23]. However, later hernias become increasingly unlikely with time—most hernias present within 24 months of surgery, with a mean delay of 19 ± 13 months [10].

Early post-operative complications are important as they may necessitate further treatment and delay discharge. In a prospective cohort study of 4565 patients, Kroese et al. reported an overall 30-day complication rate of 4.4% following primary ventral hernia repair, compared with 15% for incisional hernias (*p* < 0.001) [16]. In the present study, nine patients experienced an early seroma or ileus with no clear relation to the hernia type or use of mesh. All resolved spontaneously except for one seroma, which required aspiration. There were no wound infections. Other researchers have reported a number of major complications [13].

Although a restored QoL free from abdominal wall symptoms may be as key to procedural success as a low hernia recurrence rate for many patients, this priority has been often ignored in past research [24]. By comparison, our study focused on the PROM concept. We found that 94.5% of patients rated their outcome as “good” or “excellent” at year 2, a slight improvement from year 1 (88.3%), while patients considering their outcome “medium” or “bad” fell between years 1 and 2 (11.7% vs. 5.5%), and those considering their abdominal wall to be “firm” increased from 95.7 to 97.8%. Of 93 patients, 6 reported a lump at 12 months, and 2 reported a lump at 24 months, all in the incisional hernia group. Both lumps were in the midline and not located at the original site hernia site.

The high satisfaction rate in this study is probably related to patients’ low hernia recurrence rate and limited post-operative pain. Satisfaction did not appear to be influenced by the experiences of 12 patients reporting generally minor symptoms with no or limited effect on activity at 2 years. Liang et al. reported that 74.5% of patients were satisfied with the outcome of their laparoscopic hernia repair, and that chronic pain, changes in functional status, and recurrence were associated with reduced overall satisfaction [25]. Langbach et al. confirmed that results could be predicted by the absence of chronic pain and recurrence [26]. In the latter study, only 60.5% of patients were satisfied with their outcome after a mean of 48 months following laparoscopic ventral hernia repair, compared with 49.3% after open repair at 52 months.

Several operative factors may have contributed to the medium-term results encountered in the present study’s heterogeneous population. Guerin and Turquier previously demonstrated that the stresses applied to a piece of implanted mesh used to repair a ventral hernia are influenced by defect size and the degree of overlap with the surrounding abdominal wall [27]. Leblanc empirically recommends that there should be at least 5 cm of overlap in all directions [9]. These factors were examined further by Hauters et al., who concluded that while overlap is important, the *M*/*D* ratio is the only independent predictor of recurrence [10]. Among 16 recurrences in a cohort of 213 laparoscopic primary ventral or incisional hernia repair patients, the authors reported 70%, 35%, 9%, and 0% failure rates when the mean *M*/*D* ratio was ≤ 8, between 9 and 12, between 13 and 16, and ≥ 17, respectively.

It should be noted in the above study that the authors used a bridging technique without closing the defect. Baker et al., in a nationwide cohort from Denmark recently showed a reduced risk of reoperation for ventral hernia recurrences if the defect is closed during laparoscopic repair, in addition to the use of permanent tacks [28]. Though not universally recorded in the present study, the use of fascial closure may have contributed to the low recurrence rate to date, along with greater confidence among Club Hernie surgeons to use large pieces of mesh even for mid-sized defects. The *M*/*D* ratios of 55.7 and 22.7 for primary and incisional hernia defects in the present study are clearly larger than those reported by others [10].

Choice of mesh can influence outcomes. Liang et al. reported that polypropylene and polytetrafluoroethylene were associated with higher rates of chronic pain [25]. By comparison, implantation of low-density mesh may result in more recurrences. A multi-institutional study found that midweight mesh produced less surgical site infections and shorter hospital stays, and was associated with higher QoL scores at 6–12 months than lightweight mesh [29].

SCM, the successor to PCO, was developed with the intention of fulfilling many of the characteristics of an ideal mesh [30]. At 64 g/m^2^, it is classified as midweight and has a monofilament structure designed to encourage tissue ingrowth and mesh fixation. This feature may also reduce the risk of infection. Because SCM is relatively thin (0.7 mm), it provides flexibility, good shape memory, and ease of insertion and deployment—features of importance when using the IPOM technique. While the risk of adhesion formation has not been assessed in this study, the fact that SCM has a continuous hydrophilic film similar to PCO’s suggests that it is likely to have equivalent performances. An ultrasound examination of wound sites 12 months after PCO placement showed that 86% of patients were free of adhesions following incisional and umbilical hernia repair [12]. The success of this film was further demonstrated in a study by Chelala et al. [13]. As part of a long-term follow-up series on 1326 patients, 126 underwent further abdominal surgery for a variety of reasons, affording the opportunity to inspect for adhesions: 45.2% were adhesion free, 42.1% had loose omental adhesions, and 12.7% had easily cleavable or mild serosal adhesions to the PCO mesh.

All surgeons in this study reported that they were satisfied with the mesh’s flexibility, ease of trimming, and easy of insertion. Similarly, most were satisfied with its shape memory and low visceral attachment. Among the features that were highlighted as assisting placement were mesh transparency (74%) and orientation marking (68%). SCM appeared to be equally suitable for use in open or laparoscopic procedures, with no significant differences between approaches in the time required to position the mesh.

The strengths of the present study included an unselected cohort information being collected from both surgeons and patients from different centers, and the use of a PROM concept. Data taken from consecutive repairs of both primary and incisional hernias with either operative approach and no limitation on manner of fixation or closure of the defect represent the real-world approach of active surgeons. Further, to date there are few published studies using SCM for hernia repair.

This study did, however, have some limitations: numbers were small, there was no comparator group, patients were not physically examined at 24 months, and no objective tests were performed to assess the abdominal wall for complications. While the questionnaire is considered reliable and comparable with practices in other registries, some complications may have been missed [16, 31]. The questionnaire also screened for further repairs (Table 8); however, evidence from Helgstand et al., has shown that the reoperation rate underestimates the overall risk of recurrence by four- to fivefold [32]. This view is at odds with Hauters et al,, who used a phone interview to study outcomes among 213 patients and consider this approach sufficient for detecting recurrences accurately. They make the point that, as all patients in their study had previously experienced a hernia, they would likely be able to self-diagnose a new defect [10]. Some items in the present questionnaire (Q1, Q2, Q5) were dedicated to detecting recurrences. In the case of a positive answer, the patient was recommended to attend for clinical review. A recent study by Baucom et al. suggested that PROM may be of value in detecting recurrences [33]: The present results do at least provide evidence as to the safety, efficacy, and ease of implantation of SCM, which supports its continued use. Further research is needed to evaluate the long-term outcomes of SCM hernia repair, with randomized studies to assess how it compares to other mesh products.

## Conclusion

Repair of primary and incisional abdominal hernias using Symbotex™ composite mesh (SCM) is safe, with high patient satisfaction at 2-year follow-up. Complications were limited and non-life-threatening. There was only one questionable hernia recurrence. 12 patients reported some form of usually mild discomfort, pain, or loss of sensitivity at final assessment. These results support the long-term efficacy of SCM.
